# α-Enolase inhibits apoptosis and promotes cell invasion and proliferation of skin cutaneous melanoma

**DOI:** 10.1007/s11033-022-07540-9

**Published:** 2022-08-04

**Authors:** Kun Zhang, Ruoxi Tian, Wancong Zhang, Yishuai Li, Ning Zeng, Yan Liang, Shijie Tang

**Affiliations:** 1 Department of Plastic Surgery and Burn Center, Second Affiliated Hospital, Shantou University Medical College, Shantou, Guangdong China; 2 School of Basic Medicine, Tianjin Medical University, Tianjin, China; 3 Department of Thoracic Surgery, Hebei Chest Hospital, Shijiazhuang, Hebei China; 4 Department of Nephrology, Affiliated Hospital of Guilin Medical University, Guilin, Guangxi China

**Keywords:** ENO1, SKCM, Invasion, Proliferation, Migration

## Abstract

**Background:**

The glycolytic enzyme, α-Enolase (ENO1), catalyzes the production of phosphoenolpyruvate from 2-phosphoglycerate, thereby enhancing glycolysis and contributing to tumor progression. In the present study, we aimed to determine the role of ENO1 in skin cutaneous melanoma (SKCM) and the potential underlying mechanism.

**Methods:**

The Sangerbox database was used to analyze the mRNA expression of ENO1 in SKCM. Western blotting was used to assess the levels of ENO1, c-Myc, β-catenin, MMP-9, PGAM1, and MMP-13 in SKCM-derived cell lines or tumor tissues from patients with SKCM. The pCMV-SPORT6-ENO1 and pET-28a-ENO1siRNA plasmids were used to overexpress and knockdown ENO1 in SKCM cells, respectively. To determine the function of ENO1 in the malignant behavior of SKCM cells, we performed a wound-healing assay, cell counting kit 8 assay, and transwell chamber analyses. The production of pyruvate and lactic acid in tumor cells was evaluated using their respective kits.

**Results:**

Compared with non-tumor tissues, ENO1 was found to be overexpressed in SKCM tissues. In SKCM cells, ENO1 overexpression promoted invasion, migration, and proliferation of tumor cells; increased pyruvate and lactate production; and increased β-catenin, MMP-9, MMP-13, and c-Myc levels. The opposite effects were observed in SKCM cells silenced for ENO1.

**Conclusions:**

These results indicate that ENO1 is involved in SKCM progression by enhancing the invasion and proliferation of tumor cells. In addition, ENO1 might have an important function in tumor cell glycolysis. Therefore, ENO1 represents a potential therapeutic target for treatment of SKCM.

## Introduction

Skin cutaneous melanoma (SKCM) is the most malignant among skin cancers. In the past 30 years, its incidence has increased steadily, and it currently causes the highest skin cancer-related mortality worldwide [1]. At present, there are no effective drugs to treat patients with advanced stages of SKCM, and most patients with distant metastases succumb to the disease soon after diagnosis [2]. Increasing evidence shows that epigenetic changes are important in SKCM tumorigenesis and development. Therefore, there is a pressing need for molecular biology research to provide an experimental basis for the treatment of SKCM.

α-Enolase (ENO1, or 2-phospho-d-glycerate hydrolase) mainly catalyzes the production of phosphoenolpyruvate from 2-phosphoglycerate in glycolysis. ENO1 is multifunctional, participating in biological growth, development, and reproduction; parasitic infections; cancer occurrence and metastasis; autoantigen activity; bacterial and fungal infections; and cell stress [3]. Recent studies have found that ENO1 is highly expressed in lung adenocarcinoma, bladder cancer, pancreatic cancer, gastric cancer, colorectal cancer, and other types of tumor tissues [4–8]. While the concentration of ENO1 is important in these cancer types, little is known about the role of ENO1 in SKCM. The aim of the present study was to investigate the role of ENO1 in the pathogenesis of SKCM. This is the first study evaluating this research hypothesis.

## Materials and methods

### Sangerbox database analysis

Sangerbox (www.sangerbox.com) is a free bioinformatics tool. In this study, the Sangerbox data analysis platform was used to analyze the expression of ENO1 in SKCM and various other human tumors.

### Protein–protein interaction network analysis

STRING (https://string-db.org/) was used to build a protein–protein interaction (PPI) network selecting the options “ENO1” and “Human (Organism)”.

### GEPIA database analysis

GEPIA (http://gepia.cancer-pku.cn/) is a newly developed bioinformatics platform for analyzing transcriptome data on The Cancer Genome Atlas (TCGA) and GTEx database. We used GEPIA’s “Correlation Analysis” module to analyze the correlation between ENO1 and PGAM1 with *P* < 0.05 as the screening criterion.

### Receiver operative characteristic curve analysis

The RNAseq data from TCGA and GTEx in transcripts per million (TPM) format were processed uniformly by the Toil process of UCSC XENA (https://xenabrowser.net/datapages/). Tissue specimen data were extracted from TCGA-SKCM (n = 469) (cancerous) and GTEX (normal) (n = 813). First, log2 conversion of RNAseq data to the TPM format was performed and levels of expression were compared between the samples. The analysis was performed using R (version 3.6.3; R Foundation for Statistical Computing, Vienna, Austria) with the following packages: pROC [v.1.17.0.1 version] (for analysis) and ggplot2 [v 3.3.3] (for visualization).

On the curve, the abscissa of the curve is the false positive rate (FPR), and the ordinate is the true positive rate (TPR). The closer the area under the ROC curve (AUC) is to 1, the better the diagnostic effect. AUC has lower accuracy when it is 0.5–0.7, moderate accuracy when it is 0.7–0.9, and higher accuracy when above 0.9.

### Tissue specimens from patients with SKCM

From January 2018 to January 2020, at the Second Affiliated Hospital of Shantou University Medical College, we collected samples of SKCM tissues from 12 patients diagnosed with SKCM and naevus tissue from 12 healthy controls. Written informed consent was obtained from all the study participants. The hospital human tissue research committee approved and supervised all the procedures. The study was also approved by the Ethics Committee of Second Affiliated Hospital of Shantou University (Ethical approval number: 2021-117).

### Cell lines and culture

The Shanghai Cell Bank of the Chinese Academy of Sciences (Shanghai, China) provided four SKCM cell lines SK-MEL-28, HMCB, A375, and SK-MEL-19, which were grown in Dulbecco’s modified Eagle’s medium (Gibco® BRL; Thermo Fisher Scientific, Gaithersburg, MD, USA) in addition to 10% fetal bovine serum (Gibco® BRL), penicillin, and streptomycin (both 100 U/mL), at 37 °C in a 5% CO_2_ humidified incubator.

### Western blotting analysis

Proteins were extracted from cells and tissues using radioimmunoprecipitation assay lysis buffer (Roche, Basel, Switzerland). A bicinchoninic acid (BCA) kit (Sigma-Aldrich Co., St. Louis, MO, USA) was used to determine the total protein concentration. Equal amounts of protein (50 µg) were separated using 10% sodium dodecyl sulfate polyacrylamide gel electrophoresis and then electrotransferred onto a polyvinylidene fluoride membrane (Roche). 5% skim milk was used to block the membranes for 2 h at 26 °C, which were then incubated with mouse monoclonal antibodies recognizing human ENO1 (1:2000; Origene Technologies, Rockville, MD, USA), phosphoglycerate mutase 1 (PGAM1) (1:2000; Santa Cruz Technology, Santa Cruz, CA, USA), matrix metalloproteinase (MMP)-9 (1: 2000; Santa Cruz Technology), MMP-13 (1: 2000; Santa Cruz Technology), β-catenin (1: 2000; Santa Cruz Technology), c-Myc (1:2000; Santa Cruz Technology), or β-actin (1:10,000; Abcam, Cambridge, MA, USA), at 4 °C overnight. The membranes were then incubated with secondary antibodies (conjugated to horseradish peroxidase), comprising anti-mouse immunoglobulin IgG, for 2 h at room temperature (1:5000; Thermo Fisher Scientific, Waltham, MA, USA). We then added enhanced chemiluminescence reagent (Thermo Fisher Scientific) to the membrane, which was then analyzed using the FluorChem® HD2 Western Blot Imaging System (Alpha INNOTEC, San Leonardo, CA, USA).

### Transfection of cells

SKCM cells were grown to the logarithmic phase and then seeded into six-well plates. We transfected A375 cells with plasmids pCMV-SPORT6-ENO1 (for ENO1 overexpression) and pLENTI-CMV-GFP/Puro (contro-vector) with the aid of Lipofectamine 2000 (Thermo Fisher Scientific), following the manufacturer’s protocol (GeneCopoeia Inc, MA, USA). We transfected SK-MEL-19 cells with plasmids pET-28a-ENO1siRNA (to silence ENO1) and pET-28a (control-siRNA). Western blotting was done to confirm the successful knockdown and overexpression of ENO1. Clonal selection was performed 72 h after transfection using 0.6 µg/mL puromycin (Sigma-Aldrich Co.). Four weeks later, we harvested the stably transfected cells for subsequent analysis.

### Cell proliferation assay

To assess the proliferation of A375 and SK-MEL-19 cells, we used a Cell Counting Kit (CCK-8) (Dojindo Laboratories, Kumamoto City, Japan). The harvested, stably transfected cells were seeded into 96-well microplates (Corning Incorporated, Corning, NY, USA) at 1 × 10^3^ cells per well. For each group, six duplicate wells were set. At various time points (0, 12, 24, 48, and 72 h), CCK-8 solution (10 µL) was added to each well and incubated for 2 h at 37 °C with 5% humidified CO_2_. A Microplate Autoreader (Bio-Tek Instruments, Winooski, VT, USA) was then used to measure the absorbance of cells at 450 nm.

### Wound-healing assay

From each group, stably transfected cells in the logarithmic phase of growth were selected and inoculated into a six-well plate. The cells were grown to 100% confluence and the cell monolayer was scratched using a 200-µL sterile micropipette tip. The cells were then cultured for 24 h in a serum-free medium. Wound closure was assessed by photographing the wound under a microscope at 0 and 48 h. For each wound, at least five fields were assessed. The migration rate was calculated as the width of the scratch at 48 h divided by the width of the same scratch at 0 h.

### Transwell chamber assay

Cells of each group were collected and combined into a single-cell suspension. The cells were suspended in a serum-free medium and 200 µL of the suspension (containing 2 × 10^4^ cells) was transferred to the upper chamber of a Transwell chamber (8 μm, Corning, Inc.) that contained Matrigel (BD Biosciences, Franklin Lakes, NJ, USA). Five hundred microliters of complete medium were placed in the lower chamber. The chamber was cultured in an incubator for 24 h; the cells with the medium present in the upper chamber were removed using a cotton swab; while the cells on the lower surface of the membrane were fixed with methanol and stained using 0.1% crystal violet. The Transwell chamber was placed under an inverted microscope and the cells in five fields of view were observed and counted.

### Measurement of pyruvate and lactic acid

Twenty-four hours after the transfection of A375 and SK-MEL-19 cells, the production of pyruvate and lactic acid was examined using a Micro pyruvate assay kit and lactic acid kit (Sigma-Aldrich Co.), respectively.

### Statistical analysis

In this study, all data analyses in the database were performed using R version 3.6.2. Wilcoxon rank-sum test and Wilcoxon signed-rank test were performed to compare the expression of ENO1 in SKCM and normal tissues. All data are shown as the mean ± standard deviation. Student’s t-test was used to analyze the statistical differences between two groups, whereas one-way analysis of variance (ANOVA) was used to compare three or more groups. Statistical significance was accepted at *P* < 0.05.

## Results

### Levels of ENO1 and PGAM1 were increased in SKCM

**Fig. 1:**
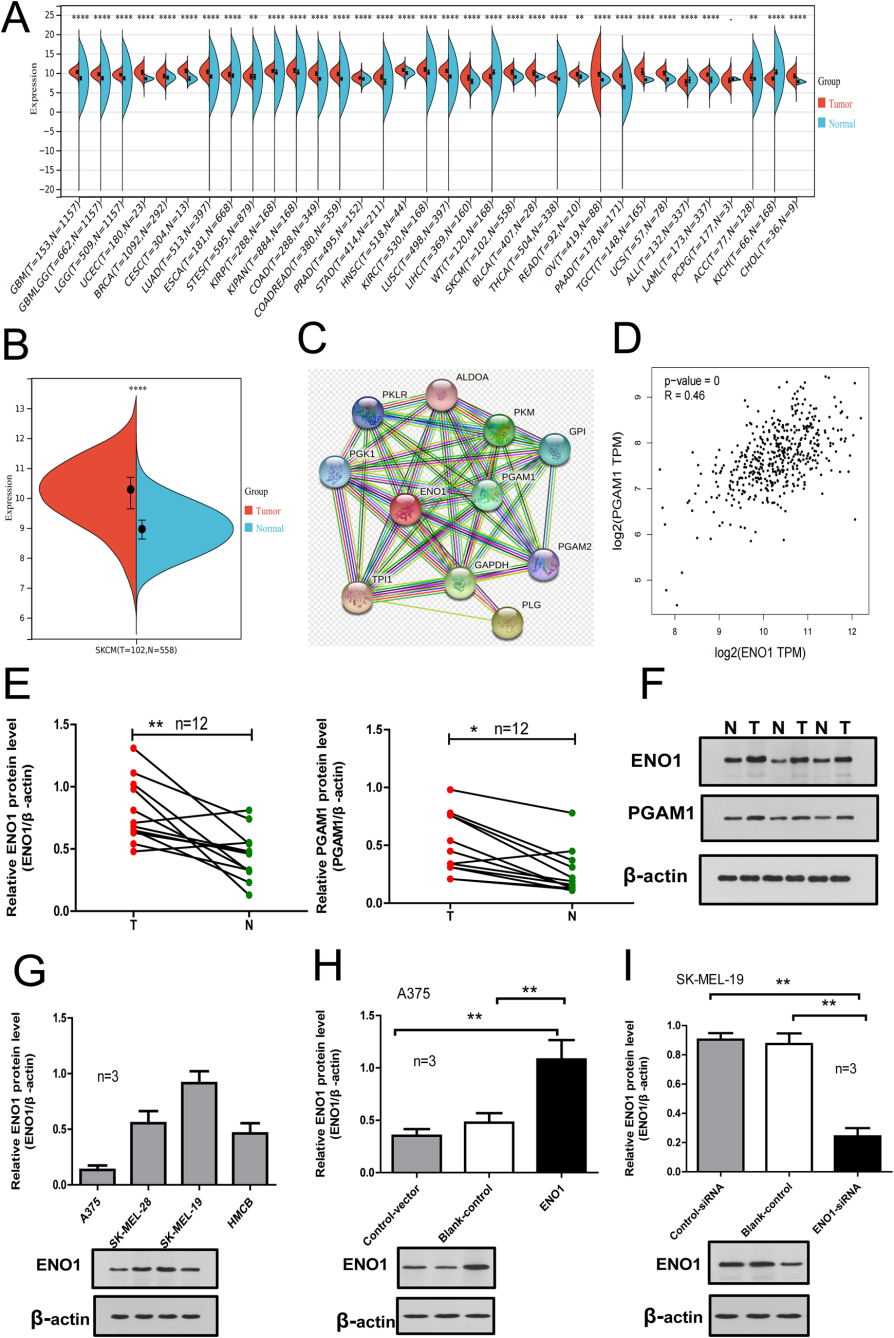
ENO1 and PGAM1 expression levels are increased in SKCM tissues and cell lines. **A** Pancreatic-cancer tissues vs. TCGA and GTEx-normal tissues. **B** SKCM tissues vs. TCGA and GTEx-normal tissues. **C** The discriminative ability of PAX3 in SKCM was evaluated by ROC curve analysis. **D** The PPI network related to ENO1, obtained from STRING datasets. **E** The correlation between ENO1 and PGAM1, obtained from the GEPIA datasets. **F**, **G** Protein levels of ENO1 and PGAM1 in SKCM, assessed using western blotting assays. T: SKCM tumor tissues; N: naevus tissues. **H** Protein levels of ENO1 in four SKCM cell lines (SK-MEL-28, HMCB, A375, and SK-MEL-19), assessed by western blotting. **I** Protein levels of ENO1 in A375 cells transfected with plasmids pCMV-SPORT6-ENO1 and pLENTI-CMV-GFP/Puro, assessed by western blotting. **J** Protein levels of ENO1 in SK-MEL-19 cells transfected with plasmids pET-28a-ENO1siRNA and pET-28a, assessed using western blotting assays. *P < 0.05, **P < 0.01. *ENO1* α-Enolase; *SKCM* skin cutaneous melanoma; *ROC* Receiver Operating Characteristic; *PGAM1* phosphoglycerate mutase 1; Protein–Protein Interaction

We used the Sangerbox platform to analyze data from TCGA and GTEx to evaluate the expression level of ENO1 in different human cancers. As shown in Fig. 1A and B, ENO1 was upregulated in multiple human tumor tissues (including SKCM). In addition, we analyzed the ROC curve to evaluate the capacity of ENO1 expression in distinguishing between SKCM and healthy subjects. As shown in Fig. 1C, the AUC was 0.786 (CI: 0.760–0.811), which implied a certain discriminative capacity of ENO1 for SKCM. We also built a PPI network using STRING and found that ENO1 functionally interacted with PGAM1 (Fig. 1D). PGAM1 was also one of the key enzymes in glycolysis. We further evaluated the correlation between ENO1 and PGAM1 based on the GEPIA database and found that ENO1 expression was positively correlated with PGAM1 in SKCM (Fig. 1E). The levels of ENO1 and PGAM1 in 12 tumor samples and nevus tissues were determined using Western blotting. The results showed that compared with that in normal benign nevus tissue samples, the levels of ENO1 and PGAM1 in SKCM tissue samples were significantly higher (Fig. 1F and G). We detected the level of ENO1 protein in human SKCM cells by Western blotting and found that it was the highest in SK-MEL-19 cells and the lowest in A375 cells (Fig. 1H). These two types of cells were used for knockdown and overexpression analysis, respectively. Western blotting was also used to verify the successful knockdown or overexpression of ENO1 (Fig. 1I and J).

### Overexpression of ENO1 promotes proliferation, invasion, and migration ofA375 cells

**Fig. 2:**
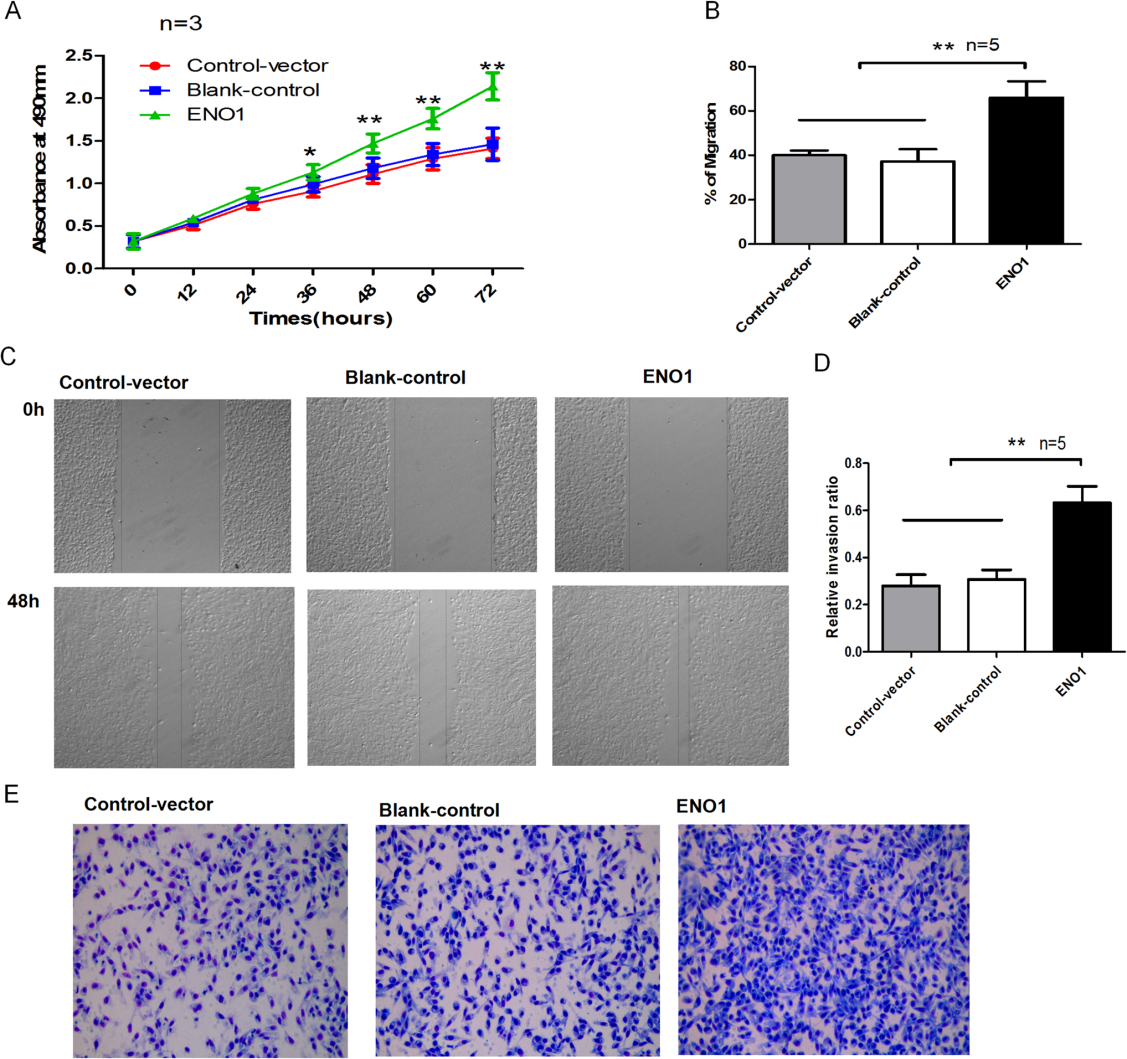
Overexpression of ENO1 inhibits apoptosis and promotes the proliferation, migration, and invasion of A375 cells. **A** CCK-8 assay showing that overexpression of ENO1 promotes the proliferation of A375 cells. **B**, **C** Wound-healing assay showing that overexpression of ENO1 increases the migration capabilities of A375 cells. **D**, **E** Transwell chamber assay showing that overexpression of ENO1 increases the invasion capabilities of A375 cells. *P < 0.05, **P < 0.01. *ENO1* α-Enolase; *CCK*-8 cell counting kit-8

PCMV-Sport6-ENO1 was transfected into A375 cells to overexpress ENO1 because of the low level of ENO1 in these cells. Plentiv-CMV-GFP/Puro (vector) was also transfected into these cells as a control. CCK-8 analysis demonstrated that after 36 h of culture, ENO1 overexpression significantly stimulated the proliferation of A375 cells than the control and blank group cells (Fig. 2A). Subsequently, we used a wound-healing assay and Transwell chamber analysis to determine the effect of overexpressing ENO1 on A375 cell migration and invasion. ENO1 overexpression increased the migration and invasion abilities of A375 cells significantly compared with that of the control cells (Fig. 2B–E). Thus, overexpression of ENO1 could promote the proliferation, invasion, and migration of SKCM cells.

### Silencing of ENO1 in SK-MEL-19 cells inhibits invasion, migration, and proliferation

**Fig. 3:**
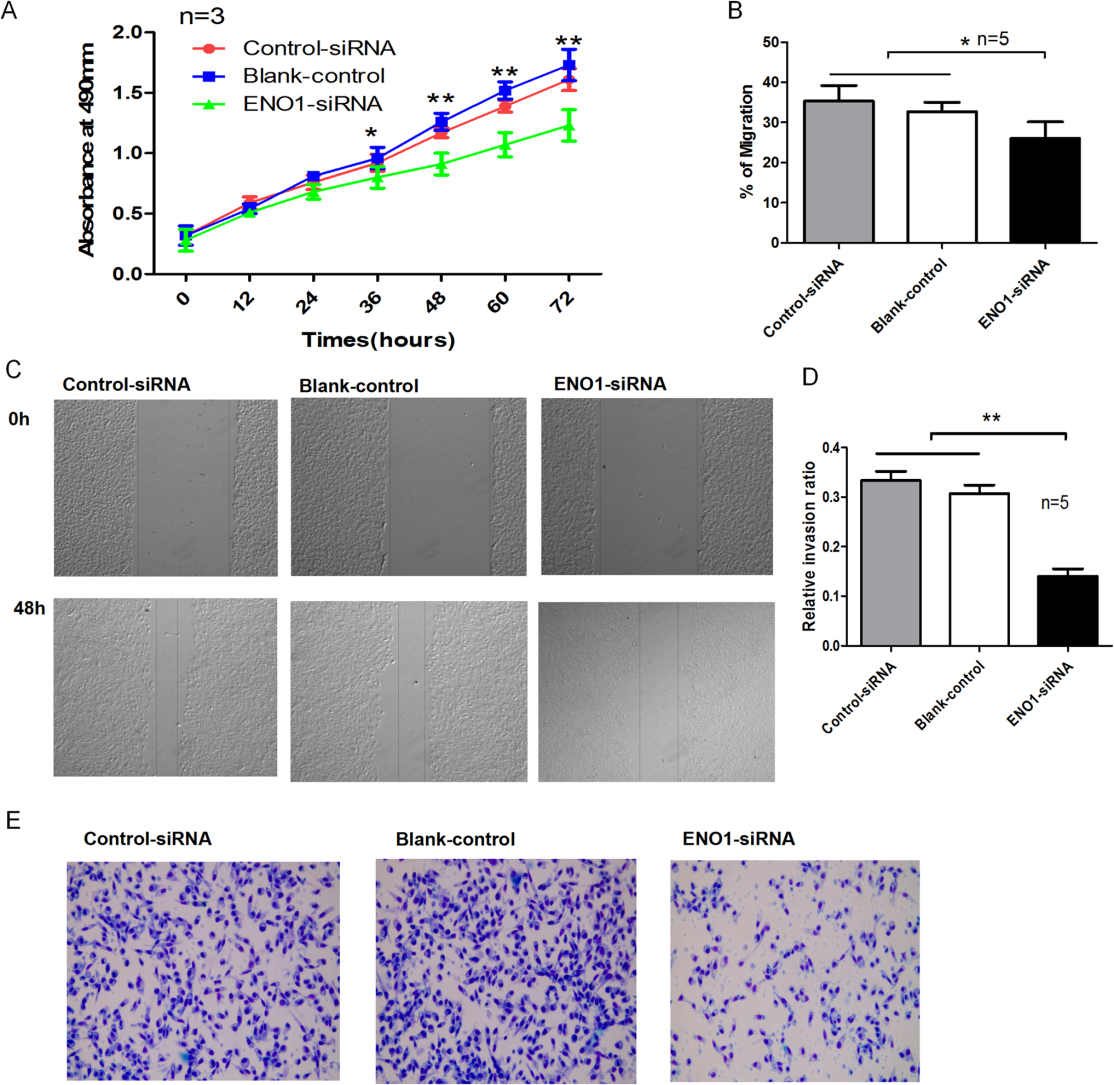
Downregulation of ENO1 induces apoptosis, and inhibits the proliferation, migration, and invasion of SK-MEL-19 cells. **A** CCK-8 assay showing that downregulation of ENO1 inhibits the proliferation of SK-MEL-19 cells. **B**, **C** Wound-healing assay showing that the downregulation of ENO1 inhibits the migration capabilities of SK-MEL-19 cells. **D**, **E** Transwell chamber assay showing that downregulation of ENO1 inhibits the invasion capabilities of SK-MEL-19 cells. *P < 0.05, **P < 0.01. *ENO1* α-Enolase; *CCK*-8 cell counting kit-8

The level of ENO1 was reduced significantly in SK-MEL-19 cells transfected with the pET-28a-ENO1siRNA plasmid CCK-8 analysis showed that the proliferation ability of ENO1*-*silenced SK-MEL-19 cells was reduced significantly at 36 h after transfection (Fig. 3A). Silencing ENO1 resulted in significant reductions in the invasion and migration abilities of SK-MEL-19 cells (Fig. 3B–E). These data indicate that silencing ENO1 expression can inhibit the invasion, migration, and proliferation of SKCM cells.

### ENO1 enhances glycolysis and increases the levels of MMP-13, MMP-9, c-Myc, and β-catenin in SKCM cells

**Fig. 4:**
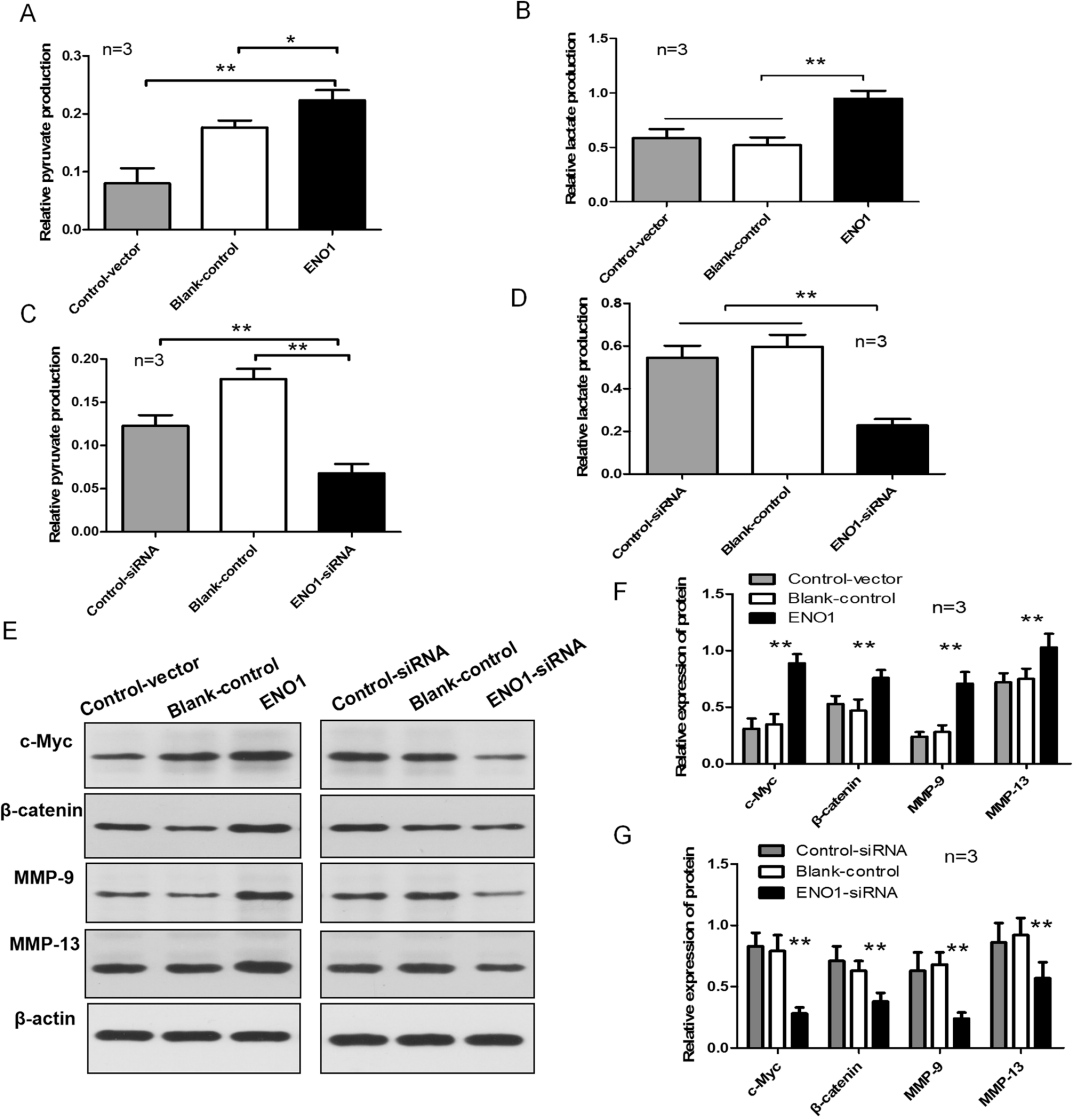
ENO1 Enhances Glycolysis and Promotes the Expression of β-catenin, c-Myc, MMP-9, and MMP-13 in SKCM cells. **A**, **B** Overexpression of ENO1 promotes the formation of lactate and pyruvate in A375 cells, measured using a micro pyruvate assay kit and lactic acid kit. **C**, **D** Downregulation of ENO1 inhibits the formation of lactate and pyruvate in SK-MEL-19 cells, assessed using a micro pyruvate assay kit and lactic acid kit. **E**, **F** Protein levels of β-catenin, c-Myc, MMP-9, and MMP-13 in A375 cells transfected with plasmid pCMV-SPORT6-ENO1 and SK-MEL-19 cells transfected with plasmid pET-28a-ENO1siRNA, assessed on western blotting assays. **P < 0.01. *ENO1* α-Enolase; *SKCM* skin cutaneous melanoma; *MMP*-9 matrix metalloproteinase-9; *MMP*-13 matrix metalloproteinase-13

ENO1 is one of the key enzymes in glycolysis, affecting the formation of key glycolytic products. Therefore, we assessed the impact of altered ENO1 expression on glycolysis in SKCM cells. ENO1 overexpression increased the levels of lactate and pyruvate in A375 cells significantly (Fig. 4A, B). By contrast, silencing ENO1 reduced the formation of lactate and pyruvate in SK-MEL-19 cells significantly (Fig. 4C, D). To determine the signaling pathway and mechanism of ENO1, the impact of altered ENO1 expression on the activity of the Wnt/β-Catenin signaling pathway and MMP-9 and MMP-13 levels was evaluated. ENO1 overexpression enhanced the levels of β-catenin and c-Myc significantly. These proteins are known to be the target of the Wnt/β-catenin signaling pathway. ENO1 overexpression also increased the levels of MMP-9 and MMP-13 significantly (Fig. 4E, F). Meanwhile, silencing ENO1 showed the opposite effects (Fig. 4E, G). Taken together, our results demonstrated that ENO1 affects the activity of the Wnt/β-catenin signaling pathway, regulates SKCM cells proliferation, and promotes their invasion and migration by increasing the levels of MMP-9 and MMP-13.

## Discussion

ENO1 mainly exists in the cytoplasm and has an important function in tumor cell glycolysis. Thus, ENO1 expression might be related to malignant tumor development [4–8]. Recently, Chinese scholars reported the production of superparamagnetic iron oxide nanoparticles targeting the highly expressed ENO1 in pancreatic cancer tissues. These nanoparticles significantly increased the diagnostic rate of pancreatic cancer by magnetic resonance imaging [9]. To the best of our knowledge, this is the first report to reveal that ENO1 and PGAM1 levels are high in SKCM cell lines and tumor tissues. Our experiments showed that ENO1 overexpression promoted the invasion, migration, and proliferation of SKCM cells.

The two main characteristics of glucose metabolism in cancer cells are: increased glucose uptake and conversion of glucose to pyruvate, which ultimately leads to the production of lactic acid (fermentation). An interesting viewpoint is that tumor glycolysis can be a potential therapeutic target [10]. Several studies have shown that the Warburg effect is one of the important signs of cancer. Warburg hypothesized that differences in energy sources are the main reason tumor cells have a higher growth rate than normal cells. Tumor cells mainly use glucose generated through glycolysis and decrease aerobic phosphorylation occurring in the mitochondria, which are considered the most important causes for tumor development [11]. If the glycolytic phenotype is reversed to oxidative phosphorylation (OXPHOS), cancer cells undergo apoptosis [12]. The rate of ATP production may be 100 times faster with glycolysis than with OXPHOS. The low yield of ATP with glycolysis is, however, sufficient to meet the intracellular demand [13]. Rapidly dividing cells such as microorganisms (with a doubling time ranging from a few minutes to several hours) require ATP for proliferation, whereas cancer cells with a comparatively longer doubling time (days rather than minutes) may require ATP primarily only for cell maintenance (rather than for proliferation). For all these reasons, the ATP formed from glycolysis is sufficient for cancer growth [13]. In our study, we found that the overexpression of ENO1 could enhance the glycolytic pathway of tumor cells. We believe that tumor progression is related to the promotion of glycolysis by ENO1.

The high expression of glycolytic enzymes might be a key factor in excessive tumor cell proliferation [14]. In addition to ENO1, PGAM1 is also highly expressed in melanoma tissues. PGAM1 is also an important glycolytic enzyme. Its main function is to catalyze the reversible production of 3-phosphoglycerate to 2-phosphoglycerate [15]. Therefore, ENO1 and PGAM1 together regulate multiple cell functions [16]. PGAM1 is highly expressed in various tumors, such as oral squamous cell carcinoma, prostate cancer, non-small cell lung cancer, renal clear cell carcinoma, pancreatic ductal carcinoma, and colorectal cancer, and is associated with tumor invasion, migration, and proliferation [17–22]. The present study showed that ENO1 and PGAM1 are highly expressed in SKCM. Understanding the cooperative mechanism by which ENO1 and PGAM1 affect SKCM development is our next research direction.

Wnt/β-catenin canonical signaling has a vital function in melanoma cell apoptosis, invasion, migration, proliferation, and differentiation [23–26]. The core component of the Wnt/β-catenin pathway, β-catenin, has an important function in tumor progression [25]. c-Myc can induce tumor cells to transition from the G1 phase to the S phase, thereby promoting cell proliferation [27]. Our study revealed that ENO1 overexpression could promote the expression of β-catenin and c-Myc significantly in SKCM cells. ENO1 knockdown, meanwhile, had the opposite result. These results indicate that the development of SKCM involves Wnt /β-catenin signaling.

We also found that ENO1 overexpression increased the formation of lactate and pyruvate in SKCM cells. During tumor growth, tumor cells must increase their glucose metabolism, and excessive proliferation under hypoxic conditions is another main feature of solid tumors [28]. The acidification of the cellular environment provides a favorable microenvironment for the activation of MMPs [29]. In almost all human malignancies, the expression level of MMPs, a class of zinc-dependent proteases, is increased, and is associated with tumor migration and invasion. The extracellular matrix (ECM), which forms a natural physical barrier that prevents tumor cells from spreading, is degraded and recombined by MMPs [30–32]. In vivo, aggressive SKCM cells show a higher expression of MMPs (especially MMP-9, MMP-10, and MMP-13) compared with the mesenchymal phenotype. MMP-9 has an important function in ECM degradation: MMP-9 activation enables the infiltration of melanoma cells into surrounding tissues and the subsequent spread of melanoma cells. The expression of MMP-9 is reportedly related to the prognosis [33–35]. The present study found that ENO1 overexpression promoted SKCM cell invasion and proliferation by altering the levels of Wnt/β-catenin-target proteins, MMP-13, MMP-9, and c-Myc. These alterations might be related to the development of SKCM caused by ENO1. Other related mechanisms and signal pathways involving ENO1 in SKCM require further exploration. This study provided only a partial theoretical basis for the treatment of SKCM.

In summary, our research confirms that ENO1, as a carcinogen responsible for melanoma, may promote tumor cell proliferation, migration, and invasion through the Wnt/β-catenin pathway, and is a potential target for the treatment of SKCM.

## Data Availability

The raw data supporting the conclusions of this article will be made available by the authors, without undue reservation.
